# Maternal suicide attempts and deaths in the first year after cesarean delivery

**DOI:** 10.1017/S0033291721005109

**Published:** 2023-05

**Authors:** Tianyang Zhang, Ängla Mantel, Bo Runeson, Anna Sidorchuk, Christian Rück, Olof Stephansson, Henrik Larsson, Zheng Chang, David Mataix-Cols, Lorena Fernández de la Cruz

**Affiliations:** 1 Centre for Psychiatry Research, Department of Clinical Neuroscience, Karolinska Institutet, Stockholm, Sweden; 2 Stockholm Health Care Services, Region Stockholm, Stockholm, Sweden; 3 Department of Women's Health, Karolinska University Hospital, Stockholm, Sweden; 4 Clinical Epidemiology Division, Department of Medicine Solna, Karolinska Institutet, Stockholm, Sweden; 5 Department of Medical Epidemiology and Biostatistics, Karolinska Institutet, Stockholm, Sweden; 6 School of Medical Sciences, Örebro University, Örebro, Sweden

**Keywords:** Maternal health, cesarean delivery, maternal death, suicide deaths, suicide attempts, postpartum period

## Abstract

**Background:**

Cesarean delivery (CD) has been associated with postpartum psychiatric disorders, but less is known about the risk of suicidal behaviors. We estimated the incidence and risk of suicide attempts and deaths during the first postpartum year in mothers who delivered via CD *v.* vaginally.

**Method:**

All deliveries in Sweden between 1973 and 2012 were identified. The mothers were followed since delivery for 12 months or until the date of one of the outcomes (i.e. suicide attempt or death by suicide), death by other causes or emigration. Associations were estimated using Cox proportional hazards regression models.

**Results:**

Of 4 016 789 identified deliveries, 514 113 (12.8%) were CDs and 3 502 676 (87.2%) were vaginal deliveries. During the 12-month follow-up, 504 (0.098%) suicide attempts were observed in the CD group and 2240 (0.064%) in the vaginal delivery group (risk difference: 0.034%), while 11 (0.0037%) deaths by suicide were registered in the CD group and 109 (0.0029%) in the vaginal delivery group (risk difference: 0.008%). Compared to vaginal delivery, CD was associated with an increased risk of suicide attempts [hazard ratio (HR) 1.46; 95% CI 1.32–1.60], but not of deaths by suicide (HR 1.44; 95% CI 0.88–2.36).

**Conclusions:**

Maternal suicidal behaviors during the first postpartum year were uncommon in Sweden. Compared to vaginal delivery, CD was associated with a small increased risk of suicide attempts, but not death by suicide. Improved understanding of the association between CD and maternal suicidal behaviors may promote more appropriate measures to improve maternal mental well-being and further reduce suicidal risks.

## Introduction

Postpartum psychiatric disorders are a major public health issue affecting up to one in four women who recently gave birth (Fawcett, Fairbrother, Cox, White, & Fawcett, 2019; Gavin et al., 2005; Shorey et al., 2018). If left unrecognized and untreated, the consequences of these disorders can be long-lasting and cause significant impairment not only to the mothers but also the child, the family, and society at large (Oates, 2003). In fact, suicide is one of the leading causes of maternal deaths (Mangla, Hoffman, Trumpff, O'Grady, & Monk, 2019; Oates, 2003), accounting for about 5–18% maternal deaths in high-income countries (Esscher et al., 2016; Grigoriadis et al., 2017; Knight et al., 2019; Lega et al., 2020; Thornton, Schmied, Dennis, Barnett, & Dahlen, 2013; Wallace, Hoyert, Williams, & Mendola, 2016) and 1–5% in low- and middle-income countries (Fuhr et al., 2014). Because psychiatric disorders are treatable and suicides are preventable, identifying potential predictors to provide adequate measures to improve maternal mental well-being and reduce maternal mortality due to suicide is of major importance.

Cesarean delivery (CD) has been reported to be associated with postpartum psychiatric disorders, such as depression, anxiety, obsessive-compulsive disorder, and post-traumatic stress disorder (Chen et al., 2020; Field, 2018; House et al., 2016; Sun, Wang, & Li, 2020). However, less is known of its association with suicidal behaviors, and the evidence from existing studies is inconclusive. Weng, Chang, Yeh, Wang, and Chen (2016) found that women who had delivered via CD had a twofold increased risk of suicide attempts, but no increased risk of deaths by suicide. Additionally, they found that postpartum depression and never having married increased the risk of postpartum suicide attempts and deaths by suicide. Another study by Schiff and Grossman (2006) did not find an association between CD and risk of postpartum suicide attempts, but a significant risk was found in the mothers who experienced fetal or infant death. However, both studies may have underestimated the occurrence of suicide attempts because only cases with a confirmed intent or cases that led to hospitalization were included. Suicide attempts that resulted in outpatient care have not been previously examined. Moreover, the association between CD and risk of suicidal behavior might be biased due to confounding by indication – namely, mothers who delivered via CD are likely to be different from those who delivered vaginally; potential differences may include sociodemographic characteristics, somatic health status, and psychiatric comorbidity. Psychiatric disorders can be an indication for CD (Admon et al., 2021) and are well-known risk factors for suicidal behaviors (Fazel & Runeson, 2020; Orsolini et al., 2016). Previous studies, however, did not address the role of psychiatric comorbidity in the association between CD and suicidal behaviors (Schiff & Grossman, 2006) or only included mood and anxiety disorders (Weng et al., 2016).

In the present Swedish national cohort study, we firstly estimated the monthly rates and cumulative incidences of suicide attempts and death by suicide during the first postpartum year, stratifying by CD *v.* vaginal delivery, and later examined the associations between CD and risk of maternal suicide attempts and deaths by suicide, while taking maternal sociodemographic characteristics and a wide range of psychiatric comorbidities into account.

## Methods

Ethical approval was obtained from the Regional Ethical Committee in Stockholm (2013/862-31/5). The requirement for informed consent was waived because the study was register-based and data on the included individuals were pseudonymized.

### Swedish national registers

Using the unique personal identification number assigned to each Swedish resident (Ludvigsson, Otterblad-Olausson, Pettersson, & Ekbom, 2009), we linked several national registers: (1) the Medical Birth Register (Axelsson, 2003), which includes data (e.g. antenatal care of the mother, the delivery record, and the record for the pediatric examination of the newborns) on more than 99% of all pregnancies and deliveries in Sweden since 1973; (2) the National Patient Register (Ludvigsson et al., 2011), which covers inpatient hospital admissions since 1969 and outpatient specialist care since 2001, with diagnoses based on the International Classification of Diseases (ICD), eighth (ICD-8; 1969–1986), ninth (ICD-9; 1987–1996), and 10th (ICD-10; 1997–2013) revisions; (3) the Total Population Register (Ludvigsson et al., 2016), which contains information about emigration since 1961 and immigration since 1969 from and to Sweden; (4) the Cause of Death Register (Brooke et al., 2017), which includes dates and causes of more than 99% of all deaths of Swedish residents, both in Sweden and abroad, since 1961; and (5) the Longitudinal Integrated Database for Health Insurance and Labour Market Studies, which covers data on education, labor, and income for all the adult population aged 16 years or older since 1990.

### Cohort and variables

We selected a cohort including all deliveries resulting in live births (from 22 gestational weeks and onwards) and stillbirths (until August 2008 from 28 gestational weeks and thereafter from 22 gestational weeks) between 1 January 1973 and 31 December 2012 from the Medical Birth Register. The corresponding mothers were followed for 12 months from the date of delivery until the date of a suicide attempt, death by suicide, death by causes other than suicide or emigration, whichever came first.

The main exposure was CD, recorded at birth and extracted from the Medical Birth Register. We stratified CDs into planned or intrapartum, depending on whether it was performed before or after labor onset, respectively. Because this variable was only available since 1990, the corresponding analyses were performed in a sub-cohort of mothers who delivered after 1990. Similarly, we divided vaginal delivery into spontaneous or instrumental (i.e. use of forceps and/or vacuum extraction) (Hauck, Lewis, Nathan, White, & Doherty, 2015).

The outcomes were maternal suicide attempts and deaths by suicide during the first postpartum year, indicated by ICD diagnostic codes. We focused on the outcomes during this period because the World Health Organization defines maternal death and late maternal death as the death of a woman while pregnant or up to one year after termination of pregnancy (World Health Organization, 2012). Data on suicide attempts were retrieved from the National Patient Register and deaths by suicide were retrieved from the Cause of Death Register. To avoid underestimation of suicidal rates and to be consistent with previous suicide research (Fernández de la Cruz et al., 2017; Haglund et al., 2014), we included both certain and undetermined causes in our definitions of suicidal behaviors, as follows: ‘suicide and self-inflicted injury’ (ICD-8 and ICD-9 codes E950–E959); ‘intentional self-harm’ (ICD-10 codes X60–X84); ‘injury undetermined whether accidental or purposely inflicted’ (ICD-8 and ICD-9 codes E980–E989); and ‘events of undetermined intent’ (ICD-10 codes Y10–Y34). Methods of suicidal behaviors were classified according to ICD grouping codes (online Supplementary eTable 1).

We included a list of covariates, namely calendar year at delivery, maternal age at delivery, maternal highest educational level (as a proxy for socioeconomic status), mother's country of birth (Sweden or outside), marital status (until 1982) or cohabitation status (after 1982), parity, previous suicide attempt, and maternal psychiatric history. Psychiatric history referred to diagnoses made prior to delivery and included anxiety and stress-related disorders, depressive and other mood disorders, substance use disorders, personality disorders, schizophrenia and other psychotic disorders, bipolar disorders, eating disorders, obsessive-compulsive disorder, attention-deficit/hyperactivity disorder, autism spectrum disorders, and intellectual disability (online Supplementary eTable 2).

### Statistical analyses

Maternal characteristics at delivery were compared between mothers who delivered via CD and those who delivered vaginally. Cumulative incidences of suicide attempts and deaths by suicide during the first postpartum year were calculated and plotted in Kaplan–Meier curves under the assumption of no competing risks, stratifying by CD or vaginal delivery. Monthly incidence rates were also calculated. The specific methods of suicidal behavior were compared between CD and vaginal delivery.

Cox proportional hazard regression models were applied to estimate hazard ratios (HRs) and corresponding 95% confidence intervals (CIs), with days since the index delivery as the underlying time scale. Estimates adjusted for calendar year of delivery (henceforth ‘minimally-adjusted’) were presented for the risk of suicide attempt or death by suicide for any CD, and intrapartum or planned CD, compared to vaginal delivery. Cluster-robust standard errors were calculated to account for the non-independence of multiple deliveries from the same mother. Additionally, because previous studies showed a significantly higher risk of suicidal behaviors in mothers at younger age, born outside of Sweden, and with history of psychiatric disorders (Esscher et al., 2016; Mangla et al., 2019; Orsolini et al., 2016), we hypothesized that maternal age, country of birth, and history of a psychiatric disorder may modify – rather than confound – the association between CD and suicidal behaviors. To assess potential effect modification, we repeated the minimally-adjusted analyses in stratified subgroup analyses and reported stratum-specific estimates. Identifying effect modifiers has the potential to provide valuable insights for targeting preventive interventions. We considered a variable an effect modifier if the stratum-specific estimates differed from each other (tested for interactions, *p* value <0.05 was considered significant) and if the overall minimally-adjusted estimate lied between them. In the final models, we adjusted for the remaining covariates and any interaction term for the exposure and effect modifiers. Missing data were imputed using the mean value for each variable. Moreover, to compare the risk of suicide attempts and deaths by suicide in the CD group and the vaginal delivery group, monthly incidence rate ratios (IRRs) were estimated using log linear Poisson regression models, comparing exposed person time (in mothers who delivered via CD) to unexposed person time (in mothers who delivered vaginally).

Four sensitivity analyses were performed. First, to assess whether instrumental vaginal delivery played a role in the associations between CD and suicidal behaviors, we repeated the Cox regression models comparing CD to spontaneous vaginal delivery. Second, we excluded women who had a stillbirth or infant loss during the first postpartum year because such tragic events may lead to an increased risk of suicidal behaviors in its own right (Qin & Mortensen, 2003; Weng et al., 2018). Third, since the National Patient Register only includes outpatient data since 2001, we analyzed the risk of suicide attempts and deaths by suicide separately in mothers who delivered before or from 2001 to examine the possible risk variation related to the changes in register coverage. Fourth, we excluded those who had a history of suicide attempt because they may be at a higher risk of attempting suicide again in the first postpartum year.

Data management and statistical analyses were done using SAS software, version 9.4 (SAS Institute) and R, version 4.0.3 (R Foundation).

## Results

### Descriptive statistics

**Fig. 1.**
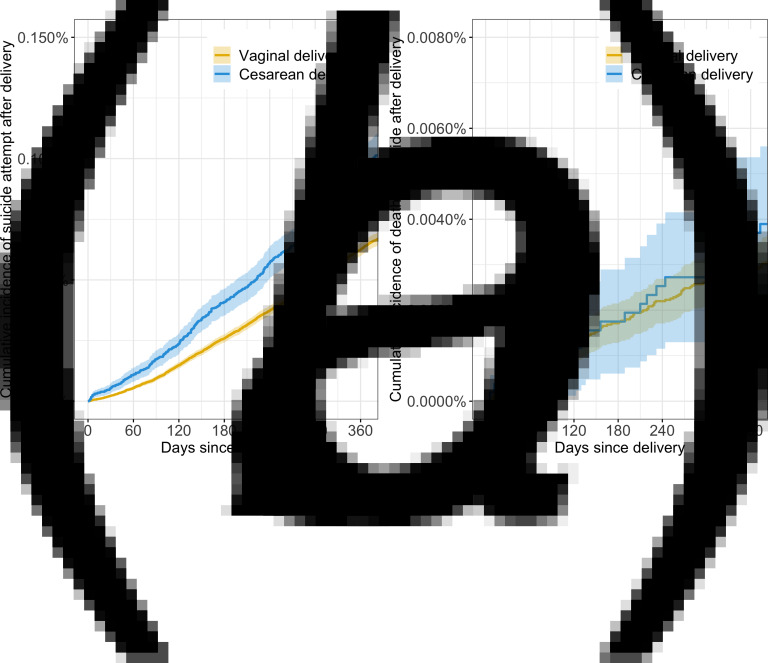
Cumulative incidence of maternal suicide attempt (*a*) and death by suicide (*b*) during the first postpartum year in mothers who delivered via cesarean delivery and vaginally.

We identified 4 016 789 index deliveries (corresponding to 2 058 674 unique mothers) among all 4 066 211 infants (including multiple gestations) who were born in Sweden between 1973 and 2012, of which 514 113 (12.8%) were CDs and 3 502 676 (87.2%) were vaginal deliveries. Mothers who delivered via CD were generally older, more likely to be foreign-born, or have a history of psychiatric disorder (Table 1). Descriptive statistics of intrapartum or planned CD and spontaneous or instrumental vaginal delivery are reported in online Supplementary eTable 3. During the 12-month follow-up after delivery, 504 (cumulative incidence 0.098%; 95% CI 0.089–0.106%) cases of suicide attempts were observed in the CD group and 2240 (cumulative incidence 0.064%; 95% CI 0.061–0.067%) in the vaginal delivery group (risk difference: 0.034%), totaling a prevalence of 0.07% (Fig. 1*a*). Nineteen deaths by suicide (cumulative incidence 0.0037%; 95% CI 0.0020–0.0054%) were registered in the CD group and 101 (cumulative incidence 0.0029%; 95% CI 0.0023–0.0035%) in the vaginal delivery group (risk difference: 0.008%), totaling a prevalence of 0.003% (Fig. 1*b*). Among those who attempted suicide or died by suicide, the specific methods used did not differ by delivery mode (online Supplementary eTable 4).

**Table 1.** Descriptive characteristics of mothers who delivered via cesarean delivery or vaginally

	All mothers, number (%)	
	Cesarean delivery (*n* = 514 113)	Vaginal delivery (*n* = 3 502 676)
*Maternal characteristics at the index delivery*		
Age, mean (s.d.)	30.2 (5.5)	28.3 (5.2)
Age groups		
<20 years	10 200 (2.0)	115 379 (3.3)
20–35 years	413 260 (80.4)	3 064 812 (87.5)
Over 35 years	90 650 (17.6)	322 480 (9.2)
Missing	3 (<0.001)	8 (<0.001)
Country of birth		
Sweden	425 303 (82.7)	2 963 794 (84.6)
Outside Sweden	88 773 (17.2)	542 500 (15.4)
Missing	37 (0.007)	304 (0.009)
Highest level of education		
Elementary education (⩽9 years)	57 774 (11.2)	471 333 (11.9)
Secondary education (10–12 years)	221 448 (43.0)	1 553 695 (44.3)
Higher education (>12 years)	209 295 (40.9)	1 361 402 (38.9)
Missing	25 596 (4.9)	170 246 (4.9)
Marital status/cohabitation		
Married (1973-1981)	49 081 (59.4)	474 200 (59.0)
Cohabiting with child's father (1982–2012)	375 497 (87.0)	2 373 629 (87.9)
Missing	38 973 (7.6)	255 194 (7.3)
Parity		
1	247 420 (48.1)	1 461 122 (41.7)
2	166 169 (32.3)	1 304 212 (37.2)
⩾3	100 524 (20.0)	737 342 (21.1)
Previous suicide attempt^a^	10 895 (2.1)	51 697 (1.5)
Maternal psychiatric history^b^	25 667 (5.0)	106 070 (3.0)
Anxiety and stress-related disorders	14 108 (2.7)	53 033 (1.5)
Depressive and other mood disorders	8275 (1.6)	31 691 (0.9)
Substance use disorders	6556 (1.3)	29 742 (0.8)
Personality disorders	3016 (0.6)	12 002 (0.3)
Schizophrenia and other psychotic disorders	1581 (0.3)	6545 (0.2)
Bipolar disorders	1237 (0.2)	4883 (0.1)
Eating disorders	1113 (0.2)	5540 (0.2)
Obsessive-compulsive disorder	699 (0.1)	2736 (0.1)
Attention-deficit/hyperactivity disorder	686 (0.1)	2737 (0.1)
Autism spectrum disorders	131 (0.03)	637 (0.02)
Intellectual disability	344 (0.1)	1376 (0.04)

s.d., standard deviation.

a History of suicide attempt prior to the index delivery.

b Included any psychiatric diagnosis made before the index delivery.

### Incidence of maternal suicidal behaviors during the first postpartum year

**Fig. 2.**
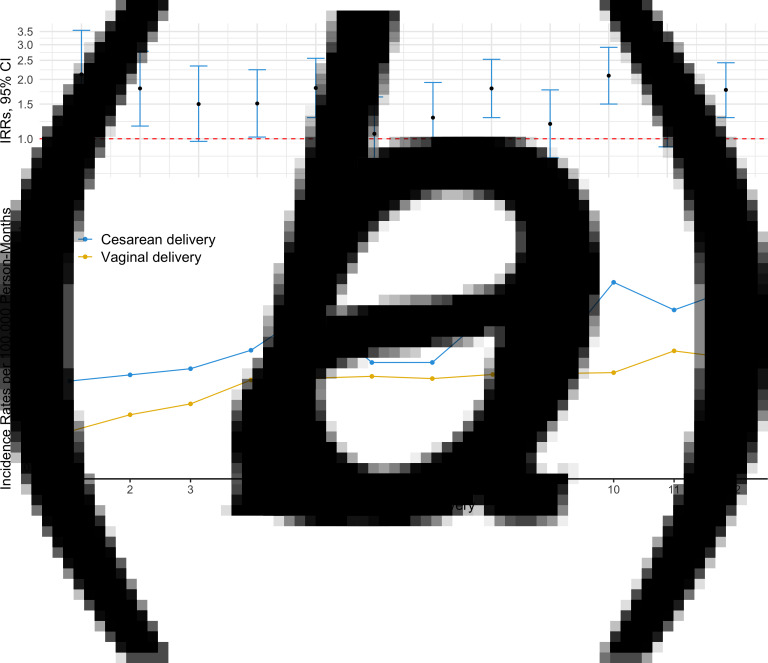
Adjusted incidence rate ratios (*a*) and incidence rates (*b*) for maternal suicide attempt by mode of delivery during the first postpartum year. CI, confidence intervals; IRRs, incidence rate ratios. Adjusted incidence rate ratios (*a*) were calculated using log linear Poisson regression models comparing mothers who delivered via cesarean delivery to mothers who delivered vaginally, adjusting for calendar year at delivery, and maternal age at delivery, highest level of education, marital status/cohabiting, parity, history of suicide attempts, history of any psychiatric disorder, and an interaction term for CD and country of birth. Incidence rates (*b*) were calculated per 100 000 person-months and stratified by mode of delivery.

During the 12-month follow-up after delivery, the monthly incidence of suicide attempt was lowest at the first postpartum month (5.45 and 2.26 per 100 000 person-months in mothers who delivered via CD or vaginally, respectively) and was highest in the final quarter (11.70 and 7.36 per 100 000 person-months in mothers who delivered via CD or vaginally, respectively) (Fig. 2*b*). Monthly incidence of death by suicide ranged from 0 to 0.78 and 0.03 to 0.40 per 100 000 person-months in mothers who delivered via CD or vaginally, respectively (online Supplementary eTable 5).

### Risk of maternal suicidal behavior during the first postpartum year

Compared to vaginal delivery, CD was associated with an increased risk of suicide attempts (HR 1.46; 95% CI 1.32–1.60). Similarly, increased risk was observed among mothers who delivered via intrapartum CD (HR 1.33; 95% CI 1.13–1.56) or planned CD (HR 1.56; 95% CI 1.34–1.82). In the stratified subgroup analyses, we found that the association between CD and suicide attempts was only significant for the mothers younger than 20 years old at delivery (HR 2.22; 95% CI 1.58–3.13), between 20 and 35 years of age (HR 1.51; 95% CI 1.35–1.68) or born in Sweden (HR 1.56; 95% CI 1.40–1.74), but became non-significant for the mothers above 35 years of age (HR 1.29; 95% CI 0.96–1.73) or foreign-born (HR 1.12; 95% CI 0.91–1.38). The estimates remained similar for both those who had a history of any psychiatric disorder (HR 1.38; 95% CI 1.18–1.62) and those that did not (HR 1.27; 95% CI 1.12–1.44). The results of the stratified subgroup analyses on the associations between intrapartum or planned CD and suicide attempts were similar to those in the corresponding analyses for any CDs, except for not having a history of any psychiatric disorder in the intrapartum CD group, which was non-significant (HR 1.18; 95% CI 0.95–1.46) (Table 2). Although the estimates differed when stratified by maternal age or country of birth, interaction tests suggested that only country of birth, but not maternal age, was likely to be an effect modifier.

**Table 2.** Risk of maternal suicidal behaviors during the first postpartum year in mothers who delivered via cesarean delivery, compared with mothers who delivered vaginally

	Minimally-adjusted hazard ratio (95% CI)^a^		
	Any CD	Intrapartum CD^b^	Planned CD^b^
*Suicide attempt*			
Compared with any VD	**1.46 (1.32–1.60)**	**1.33 (1.13–1.56)**	**1.56 (1.34–1.82)**
*Stratified subgroup analyses*			
Age groups			
<20 years	**2.22 (1.58–3.13)**	**1.94 (1.23–3.05)**	**2.48 (1.51–4.09)**
20–35 years	**1.51 (1.35–1.68)**	**1.31 (1.09–1.58)**	**1.77 (1.50–2.10)**
Over 35 years	1.29 (0.96–1.73)	1.36 (0.84–2.21)	1.07 (0.67–1.70)
Country of birth^c^			
Sweden	**1.56 (1.40–1.74)**	**1.38 (1.14–1.66)**	**1.78 (1.45–2.04)**
Outside Sweden	1.12 (0.91–1.38)	1.16 (0.83–1.61)	1.11 (0.78–1.57)
History of any psychiatric disorder			
Yes	**1.38 (1.18–1.62)**	**1.38 (1.06–1.78)**	**1.41 (1.11–1.78)**
No	**1.27 (1.12–1.44)**	1.18 (0.95–1.46)	**1.37 (1.12–1.67)**
*Death by suicide*			
Compared with any VD	1.44 (0.88–2.36)	1.48 (0.58–3.76)	0.91 (0.28–2.96)
*Stratified subgroup analyses*			
Age groups			
<20 years	NR^d^	NR^d^	NR^d^
20–35 years	1.29 (0.72–2.32)	1.05 (0.32–3.42)	1.18 (0.36–3.83)
Over 35 years	2.32 (0.84–6.39)	5.27 (0.88–31.65)	NR^d^
Country of birth^c^			
Sweden	1.03 (0.55–1.93)	1.15 (0.35–3.75)	0.76 (0.18–3.20)
Outside Sweden	**3.15 (1.36–7.27)**	2.60 (0.55–12.24)	1.48 (0.18–11.89)
History of any psychiatric disorder			
Yes	1.52 (0.68–3.39)	1.91 (0.55–6.63)	0.51 (0.07–3.90)
No	1.17 (0.62–2.21)	1.00 (0.24–4.22)	1.08 (0.26–4.59)

CD, cesarean delivery; CI, confidence interval; NR, not relevant; VD, vaginal delivery.

a Model-adjusted for calendar year at delivery.

b Analyses were performed in a sub-cohort of women who delivered between 1990–2012.

c *p* Value for interaction test <0.05.

d Hazard ratio was not calculated because there were no exposed cases in this group.

*P*-value <0.05.

In contrast, mothers who delivered via CD were not at higher risk of deaths by suicide (HR 1.44; 95% CI 0.88–2.36), including when divided into intrapartum (HR 1.48; 95% CI 0.58–3.76) or planned CD (HR 0.91; 95% CI 0.28–2.96), compared to those who delivered vaginally. However, we observed a significantly higher risk of deaths by suicide when comparing the whole CD group to vaginal delivery group among mothers who were born outside Sweden (HR 3.15; 95% CI 1.36–7.27), and the interaction test suggested effect modification by this variable (Table 2).

In the fully adjusted models where we accounted for confounders and an interaction term for CD and country of birth, the risk of suicide attempts was overall unchanged (overlapping CIs) in the CD [adjusted HR (aHR) 1.57; 95% CI 1.41–1.75], intrapartum CD (aHR 1.47; 95% CI 1.22–1.78), and planned CD (aHR 1.74; 95% CI 1.47–2.06) groups, compared to vaginal delivery. The risk of death by suicide remained non-significant in the CD (aHR 0.81; 95% CI 0.43–1.53), intrapartum CD (aHR 0.91; 95% CI 0.27–2.99), and planned CD (aHR 0.66; 95% CI 0.16–2.78) groups, compared to vaginal delivery (online Supplementary eTable 6).

The monthly incidence rate of suicide attempts was significantly higher in mothers who delivered via CD, compared to vaginal delivery, in the first (IRR 2.12; 95% CI 1.26–3.55), second (IRR 1.80; 95% CI 1.16–2.78), fourth (IRR 1.51; 95% CI 1.02–2.24), fifth (IRR 1.81; 95% CI 1.28–2.56), eighth (IRR 1.80; 95% CI 1.28–2.53), 10th (IRR 2.09; 95% CI 1.50–2.91), and 12th (IRR 1.77; 95% CI 1.28–2.43) months postpartum (Fig. 2*a*). The IRRs for death by suicide were not calculated due to the small number of cases.

We next conducted four sensitivity analyses. The first analysis showed that instrumental vaginal delivery was not associated with risk of suicide attempts or deaths by suicide, compared to spontaneous vaginal delivery. Moreover, the association between CD and suicide attempts or deaths by suicide did not differ when instrumental delivery was excluded (online Supplementary eTable 7). Second, when mothers who had a stillbirth or infant loss during the first postpartum year were excluded (*n* = 34 139), the pattern of results remained largely unchanged for the association between CD and suicide attempt (aHR 1.55; 95% CI 1.39–1.74) and deaths by suicide (aHR 0.71; 95% CI 0.36–1.43). Third, stratifying the analyses into births before or from 2001 resulted in similar estimates (overlapping CIs) both for suicide attempts for the births before 2001 (aHR 1.60; 95% CI 1.38–1.87) and from 2001 (aHR 1.47; 95% CI 1.25–1.73), and for death by suicide for the births before 2001 (aHR 0.88; 95% CI 0.42–1.84) and from 2001 (aHR 0.65; 95% CI 0.19–2.26). Fourth, excluding women with history of suicide attempts did not change the associations between CD and suicide attempt (aHR 1.54; 95% CI 1.35–1.75) nor death by suicide (aHR 0.81; 95% CI 0.40–1.63).

## Discussion

This large nationwide cohort study including over 4 million deliveries estimated the incidence and the risk of maternal suicidal behaviors in the first postpartum year in relation to mode of delivery. Four main sets of conclusions can be derived from our results.

First, maternal suicidal behaviors during the first postpartum year were uncommon in Sweden. The rate of deaths by suicide (0.003%) in our study was lower than the rate in the general female population in reproductive age (15–49 years) in Sweden (ranging from 0.009% to 0.014% from year 1990 to 2012) (Institute for Health Metrics and Evaluation, 2015). This suggests that there might be a protective effect of recent delivery against suicidal behaviors, as suggested by other studies (Appleby, 1991; Lindahl, Pearson, & Colpe, 2005; Mota et al., 2019). Our estimate for suicide attempts (0.07%) is comparable with a register-based study conducted in Canada (Mota et al., 2019), which reported a rate of 0.06%. However, our estimate was lower than those in studies conducted in China (0.14%, hospital-based) (Yang, 2007), the USA (0.2%, hospital-based) (Admon et al., 2021), and Zimbabwe (4%, self-reported) (Shamu, Zarowsky, Roelens, Temmerman, & Abrahams, 2016), and much higher than that in a Taiwanese study (0.009%, hospital-based) (Weng et al., 2016). Factors contributing to the discrepancies may include heterogeneity in the settings, data collection, study designs, and sample sizes. On the other hand, our estimate of deaths by suicide (0.003%) is overall comparable with estimates from other high-income countries, including the USA (0.0016–0.0045%) (Wallace et al., 2016), Canada (0.003%) (Grigoriadis et al., 2017), and Finland (0.006%) (Gissler, Hemminki, & Lonnqvist, 1996), despite some variations.

Second, we observed a notable peak of suicide attempts in the final quarter of the first postpartum year, regardless of delivery mode, which is consistent with previous findings (Grigoriadis et al., 2017; Schiff & Grossman, 2006; Thornton et al., 2013). Moreover, compared to mothers who delivered vaginally, the incidence rates of suicide attempts were significantly higher in mothers who delivered via CD in several months across the first postpartum year. In Sweden, mothers receive standard postnatal care immediately after birth (Hildingsson, 2007). Since 2010, a general screening program for depression has been implemented for all new mothers at the time when the child is 6–8 weeks old. Our results indicate that a later contact or screening may be motivated. In some Swedish regions, a follow-up interview is offered when the child is 8–12 months old (Bränn et al., 2021), which may be an initiative to implement at the national level. Because at no other time do women have more contact with the healthcare system, the postpartum period may represent a golden opportunity to detect early signs of mental health problems (Esscher et al., 2016; Khalifeh, Hunt, Appleby, & Howard, 2016; Mangla et al., 2019).

Third, our adjusted analyses showed that CD was associated with a 46% increased risk of suicide attempts. This finding is in the same direction but of a smaller magnitude than that in a previous, smaller study with 139 identified suicide attempts in Taiwan (adjusted odds ratio 2.38; 95% CI 1.56–3.62) (Weng et al., 2016). It is worth noting that the CD rate in Taiwan (about 30%) was much higher than in our cohort (13%), because many CDs performed there were upon maternal request (Chu et al., 2015; Chu, Tai, Hsu, Yeh, & Chien, 2010), whereas in Sweden CDs were most likely performed in the presence of a medical indication (Socialstyrelsen, 2019; Wiklund, Andolf, Lilja, & Hildingsson, 2012). Women who request CDs are more likely to be psychologically vulnerable (Eide, Morken, & Bærøe, 2019; Matinnia et al., 2018), which could predispose to mental illness and suicidality. At the same time, intrapartum CDs may lead to psychological distress, particularly post-traumatic stress conditions (Benton, Salter, Tape, Wilkinson, & Turnbull, 2019; Chen et al., 2020), which may increase the risk of suicidal behaviors. However, we did not find a significant difference in the risk estimates between intrapartum (42% increased risk) and planned (59% increased risk) CD. Future studies are needed to investigate the associations between specific indications for CDs and suicidal behaviors. In line with previous studies (Lysell et al., 2018; Weng et al., 2016), we did not find an association between CD and death by suicide, although it should be noted that the analyses may be underpowered due to maternal death by suicide being a rare event in our study (120 deaths in over 4 million deliveries).

Fourth, our subgroup analyses showed that the magnitude of the association between CD and suicide attempt was particularly notable in women aged below 20 years at delivery. Moreover, CD was only associated with suicide attempt among mothers born in Sweden, but not in foreign-born mothers. This contrasts with the deaths by suicide, where a threefold increased risk was observed among foreign-born mothers when comparing CD *v.* vaginal delivery. This might reflect the lower likelihood of seeking mental health care among foreign-born mothers due to language barriers and mental illness stigma (Wangel et al., 2012), rather than an actual lower risk of suicide attempt. Sweden is a multi-ethnic society, and about every sixth birth delivered in our study was by mothers born abroad. A better mental health management of foreign-born mothers may be relevant.

Our study has several strengths, including the linkage of several large nationwide registers that hold prospectively collected data, the high statistical power owing to the four-decade study period, and a careful analytical strategy accounting for potential confounding and effect modification, particularly the impact of a wide range of psychiatric disorders and history of suicide attempts. Another strength was the inclusion of undetermined suicide attempt and suicide deaths to avoid underestimation of suicide cases (Fernández de la Cruz et al., 2017; Haglund et al., 2014). Nonetheless, our conclusions should be interpreted in light of some limitations. First, we did not explore the impact of some factors that may confound the association between CD and suicidal behaviors, such as physical illness (Kaplan, McFarland, Huguet, & Newsom, 2007; Qin, Hawton, Mortensen, & Webb, 2014), early-life adversity (Fazel & Runeson, 2020; Orsolini et al., 2016), intimate partner violence (Akaba & Abdullahi, 2020; Devries et al., 2013; Shamu et al., 2016), and lack of contact with psychiatric care or discontinuation of treatments (Khalifeh et al., 2016; Mangla et al., 2019). The small increased risk of suicide attempts in mothers following CD in the present study, together with the potential impact of unmeasured confounders, calls for cautious interpretation of the findings and the need for replication. Second, the National Patient Register, where we obtained data on suicide attempts and diagnoses of psychiatric disorders, only included outpatient data from 2001. As a result, we may have only captured cases of suicide attempts with higher lethality or psychiatric disorders in the more severe end of the spectrum. Nevertheless, the association between CD and suicide attempts still held in our sensitivity analysis focusing only the later period including both in- and outpatient outcomes. Last, it is unclear if our findings can be generalized to other settings owing to disparities in culture, health policy, and healthcare system.

## Conclusions

Maternal suicidal behaviors during the first postpartum year were uncommon in Sweden. Compared to vaginal delivery, CD was associated with a small but statistically significant increased risk of suicide attempts during the first postpartum year, particularly among younger mothers and Sweden-born mothers. We did not find an association between CD and deaths by suicide, although this association became significant for foreign-born mothers in a sub-group analysis. A better understanding of the association between CD and maternal suicidal behaviors may promote more appropriate measures to improve maternal mental well-being and further reduce suicidal risks.

## Supplementary material

For supplementary material accompanying this paper visit https://doi.org/10.1017/S0033291721005109. click here to view supplementary material
