# Supplementary material for: Maternal suicide attempts and deaths in the first year after cesarean delivery
Source: Psychol Med. 2022 Dec 16;53(7):3056–64. doi: 10.1017/S0033291721005109 (PMC10235649; doi:10.1017/S0033291721005109)
Supplement: Supplementary file 1 [file S0033291721005109sup001.docx]

**Supplemental Material**

**Maternal suicide attempts and deaths in the first year after cesarean delivery**

Tianyang Zhang^1,2^, Ängla Mantel^3,4^, Bo Runeson^1,2^, Anna Sidorchuk^1,2^, Christian Rück^1,2^, Olof Stephansson^3,4^, Henrik Larsson^5,6^, Zheng Chang^5^, David Mataix-Cols^1,2^, Lorena Fernández de la Cruz^1,2^

^1^ Centre for Psychiatry Research, Department of Clinical Neuroscience, Karolinska Institutet, Stockholm, Sweden
^2^ Stockholm Health Care Services, Region Stockholm, Stockholm, Sweden

^3^ Department of Women’s Health, Karolinska University Hospital, Stockholm, Sweden

^4^ Clinical Epidemiology Division, Department of Medicine Solna, Karolinska Institutet, Stockholm, Sweden ^5^ Department of Medical Epidemiology and Biostatistics, Karolinska Institutet, Stockholm, Sweden

^6^ School of Medical Sciences, Örebro University, Örebro, Sweden

**Corresponding author:**

Tianyang Zhang, Karolinska Institutet, Department of Clinical Neuroscience, Child and Adolescent Psychiatry Research Center, Gävlegatan 22 (Entré B), Floor 8, SE-11330 Stockholm, Sweden, email: Tianyang.Zhang@ki.se

**CONTENTS**

**Supplemental eTable 1**. *International Classification of Diseases* (ICD) Eighth (*ICD-8*; 1967-1986), Ninth (*ICD-9*; 1987-1996), and Tenth (*ICD-10*; 1997-2013) Revisions Codes for Methods of Suicide and Attempted Suicide

**Supplemental eTable 2**. *International Classification of Diseases* (ICD) Eighth (*ICD-8*; 1967-1986), Ninth (*ICD-9*; 1987-1996), and Tenth (*ICD-10*; 1997-2013) Revisions Codes for Psychiatric Comorbidity in the Study

**Supplemental eTable 3.** Descriptive Characteristics of Mothers who Delivered via Emergent or Planned Cesarean Delivery or Assisted or Unassisted Vaginal delivery in Mothers who Delivered in Sweden 1990-2012

**Supplemental eTable 4.** Methods of Attempted Suicide and Death by Suicide Stratified by Mode of Delivery

**Supplemental eTable 5.** Monthly Incidence Rates for Death by Suicide Stratified by Mode of Delivery at Each Month During the 12-Month Follow-up

**Supplemental eTable 6.** Adjusted Risk of Maternal Suicidal Behaviors During the First Postpartum Year in Mothers who Delivered via Cesarean Delivery, Compared with Mothers who Delivered Vaginally

**Supplemental eTable 7.** Risk of Maternal Suicidal Behaviors During the First Postpartum Year in Mothers who Delivered via Cesarean Delivery, Compared with Mothers who Delivered via Unassisted Vaginal Delivery

**Supplemental eTable 1**. *International Classification of Diseases* (ICD) Eighth (*ICD-8*; 1967-1986), Ninth (*ICD-9*; 1987-1996), and Tenth (*ICD-10*; 1997-2013) Revisions Codes for Methods of Suicide and Attempted Suicide

|  | ***ICD-8^a^*** | ***ICD-9 ^a^*** | ***ICD-10 ^a^*** |
| --- | --- | --- | --- |
| **Methods of suicide** | | | |
| **Non-violent** | | | |
| Poisoning | E950, E980 | E950, E980 | X60-X65, Y10-Y15 |
| **Violent** | | | |
| Gassing | E951, E952, E981, E982 | E951, E952, E981, E982 | X66-X69, Y16-Y19 |
| Hanging, strangulation, suffocation | E953, E983 | E953, E983 | X70, Y20 |
| Drowning | E954, E984 | E954, E984 | X71, Y21 |
| Firearm or explosive | E955, E985 | E955, E985 | X72-X75, Y22-Y25 |
| Smoke, fire, and flames or steam and hot objects | - | - | X76-X77, Y26-Y27 |
| Cutting or piercing | E956, E986 | E956, E986 | X78-X79, Y28-Y29 |
| Jumping from a height | E957, E987 | E957, E987 | X80, Y30 |
| Jumping or lying before moving object | E958, E988 | E958, E988 | X81, Y31 |
| Crashing of motor vehicle | - | - | X82, Y32 |
| Other means | E959 | E989 | X83-X84, Y33-Y34 |

^a^ According to Swedish ICD codes.

**Supplemental eTable 2**. *International Classification of Diseases* (ICD) Eighth (*ICD-8*; 1967-1986), Ninth (*ICD-9*; 1987-1996), and Tenth (*ICD-10*; 1997-2013) Revisions Codes for Psychiatric Comorbidity in the Study

| **Psychiatric Comorbidity** | ***ICD Codes^a^*** |
| --- | --- |
| Anxiety and stress-related disorders | ICD-9 300A, 300C, 308, 309  ICD-10 F40, F41, F43 |
| Depression and other mood disorders | ICD-9 296B, 296X, 298A, 300E, 311  ICD-10 F32, F33, F34 (excluding F34.0), F38, F39 |
| Substance use disorders | ICD-8 303, 304  ICD-9 303, 304, 305A, 305X  ICD-10 F10-F16 and F18-19 (minus F1x.5) |
| Personality disorders | ICD-8 301  ICD-9 301  ICD-10 F60 |
| Schizophrenia and other psychotic disorders | ICD-9 295 (excluding 295F), 297, 298 (excluding 298A)  ICD-10 F20, F21, F22, F23, F24, F25 (excluding F25.0), F28, F29 |
| Bipolar disorders | ICD-9 296A, 296C, 296D, 296E, 296W, 296X  ICD-10 F25.0, F30, F31, F34.0 |
| Eating disorders | ICD-9 307B, 307F  ICD-10 F50.0-F50.3, F50.9 |
| Obsessive-compulsive disorder | ICD-9 300D  ICD-10 F42 |
| Attention-deficit/hyperactivity disorder | ICD-9 314  ICD-10 F90  Medication for ADHD ^b^: amphetamine [ATC code N06BA01], dexamphetamine [ATC code N06BA02], methylphenidate [ATC code N06BA04], atomoxetine [ATC code N06BA09], and lisdexamphetamine [ATC code N06BA12] |
| Autism spectrum disorders | ICD-9 299  ICD-10 F84.0, F84.1, F84.3, F84.5, F84.8, F84.9 |
| Intellectual disability | ICD-9 317-319  ICD-10 F70, F71, F72, F73, F78, F79 |

Abbreviations: ADHD: Attention-deficit/hyperactivity disorder; ATC: Anatomical Therapeutic Chemical; ICD: International Classification of Diseases.

^a^ According to Swedish ICD codes.

^b^ It was possible to identify all dispensed medications approved in Sweden for the management of ADHD from the Prescribed Drug Register. The listed medications were approved in Sweden for the management of ADHD during the time period relevant for the study.

**Supplemental eTable 3.** Descriptive Characteristics of Mothers who Delivered via Emergent or Planned Cesarean Delivery or Assisted or Unassisted Vaginal delivery in Mothers who Delivered in Sweden 1990-2012

|  | Mothers who delivered in Sweden 1990-2012, n (%) | | | |
| --- | --- | --- | --- | --- |
|  | Cesarean Delivery  (n=326 547) | | Vaginal Delivery  (n=1 871 265) | |
|  | Intrapartum (n=164 488) | Planned  (n=162 059) | Instrumental (n=171 461) | Spontaneous (n=1 699 804) |
| Maternal Characteristics at the Index Delivery | | | | |
| Age Groups | | | | |
| < 20 years | 2 361 (1.4) | 1 455 (0.9) | 3 285 (1.9) | 35 358 (2.1) |
| 20-35 years | 134 301 (81.6) | 121 423 (74.9) | 148 178 (86.4) | 1 463 696 (86.1) |
| Over 35 years | 27 825 (16.9) | 39 179 (24.2) | 19 996 (11.7) | 200 747 (11.8) |
| Missing | 1 (0) | 2 (0) | 2 (0) | 3 (0) |
| Country of Birth | | | | |
| Sweden | 131 152 (79.7) | 131 748 (81.3) | 141 578 (82.6) | 1 398 161 (82.3) |
| Outside Sweden | 33 326 (20.3) | 30 302 (18.7) | 29 870 (17.4) | 301 524 (17.7) |
| Missing | 10 (0) | 9 (0) | 13 (0) | 119 (0) |
| Highest Level of Education | | | | |
| Elementary education (≤9 years) | 14 739 (9.0) | 14 486 (8.9) | 11 910 (6.9) | 157 053 (9.2) |
| Secondary education (10-12 years) | 70 382 (42.7) | 68 543 (42.3) | 70 467 (41.1) | 741 285 (43.6) |
| Higher education (>12 years) | 74 456 (45.3) | 74 374 (45.9) | 84 591 (49.3) | 753 419 (44.4) |
| Missing | 4 911 (3.0) | 4 656 (2.9) | 4 493 (2.6) | 48 047 (2.8) |
| Cohabiting | | | | |
| Cohabiting with child's father | 143 523 (87.3) | 142 678 (88.0) | 151 721 (88.5) | 1 508 133 (88.7) |
| Missing | 9 758 (5.9) | 8 259 (5.1) | 9 593 (5.6) | 84 509 (5.0) |
| Parity | | | | |
| 1 | 100 242 (60.9) | 55 383 (34.2) | 134 891 (78.7) | 652 688 (38.4) |
| 2 | 42 679 (25.9) | 63 701 (39.3) | 28 109 (16.4) | 665 301 (39.1) |
| ≥ 3 | 21 567 (13.2) | 42 975 (26.5) | 8 461 (4.9) | 381 815 (22.5) |
| Previous Suicide Attempt^a^ | 4 017 (2.4) | 4 477 (2.8) | 3 112 (1.8) | 34 333 (2.0) |
| Maternal Psychiatric History^b^ | 9 736 (5.9) | 12 236 (7.6) | 8 034 (4.7) | 77 483 (4.6) |
| Anxiety and stress-related disorders | 5 313 (3.2) | 7 234 (4.5) | 4 144 (2.4) | 39 911 (2.3) |
| Depressive and other mood disorders | 3 516 (2.1) | 4 383 (2.7) | 2 824 (1.6) | 26 399 (1.6) |
| Substance use disorders | 2 579 (1.6) | 2 820 (1.7) | 2 200 (1.3) | 21 335 (1.3) |
| Personality disorders | 981 (0.6) | 1 306 (0.8) | 766 (0.4) | 7 526 (0.4) |
| Schizophrenia and other psychotic disorders | 483 (0.3) | 574 (0.4) | 385 (0.2) | 3 579 (0.2) |
| Bipolar disorders | 465 (0.3) | 536 (0.3) | 362 (0.2) | 3 322 (0.2) |
| Eating disorders | 478 (0.3) | 601 (0.4) | 501 (0.3) | 4 706 (0.3) |
| Obsessive-compulsive disorder | 246 (0.1) | 377 (0.2) | 233 (0.1) | 2 131 (0.1) |
| Attention-deficit/hyperactivity disorder | 297 (0.2) | 369 (0.2) | 244 (0.1) | 2 407 (0.1) |
| Intellectual disability | 155 (0.1) | 123 (0.1) | 110 (0.06) | 966 (0.06) |
| Autism spectrum disorders | 59 (0.04) | 63 (0.04) | 67 (0.04) | 557 (0.03) |

Abbreviation: SD, standard deviation.

^a^History of suicide attempt prior to the index delivery.

^b^Maternal psychiatric history included any psychiatric diagnosis made before the index delivery.

**Supplemental eTable 4.** Methods of Attempted Suicide and Death by Suicide Stratified by Mode of Delivery

| **Method of Attempted Suicide, n (%)**^a^ | Cesarean delivery  (n = 504) | Vaginal delivery  (n = 2 240) |
| --- | --- | --- |
| **Non-violent** |  |  |
| Poisoning | 379 (75.2) | 1633 (72.9) |
| **Violent**^b^ | 125 (24.8) | 607 (27.1) |
| Hanging, strangulation, suffocation | 5 (1.0) | 23 (1.0) |
| Cutting or piercing | 25 (5.0) | 117 (5.2) |
| Jumping from a height | 11 (2.2) | 29 (1.4) |
| Jumping or lying before moving object | 8 (1.7) | 41 (2.1) |
| Other means^c^ | 76 (15.1) | 397 (17.7) |
| **Method of Death by Suicide, n (%)**^a^ | Cesarean delivery  (n = 19) | Vaginal delivery  (n = 101) |
| **Non-violent** |  |  |
| Poisoning | 6 (31.6) | 21 (20.8) |
| **Violent**^b^ | 13 (68.4) | 80 (79.2) |
| Hanging, strangulation, suffocation | 5 (26.3) | 36 (35.6) |
| Other means^d^ | 8 (42.1) | 44 (8.9) |

^a^ P-value for Chi-square tests were all larger than 0.05.

^b^ Suicide methods with too few cases (<5) were grouped into the group of ‘other means’ because of risk of individual identification.

^c^ Including gassing, drowning, firearm or explosives, smoke, fire, and flames or steam and hot objects, crashing of motor vehicle, and other methods that were not specified in the International Classification of Diseases.

^d^ Including gassing, firearm or explosives, cutting or piercing, jumping from a height, and jumping or lying before moving object.

**Supplemental eTable 5.** Monthly incidence Rates for Death by Suicide Stratified by Mode of Delivery During the 12-Month Follow-up

| Months | Incidence rates, cases per 100,000 person-months (95% CI) | |
| --- | --- | --- |
|  | Cesarean delivery | Vaginal delivery |
| 1 | 0.39 (0.10, 1.56) | 0.29 (0.15, 0.53) |
| 2 | 0.39 (0.10, 1.56) | 0.23 (0.11, 0.46) |
| 3 | 0.19 (0.03, 1.38) | 0.31 (0.17, 0.57) |
| 4 | 0 | 0.40 (0.24, 0.68) |
| 5 | 0.58 (0.19, 1.81) | 0.31 (0.17, 0.57) |
| 6 | 0.19 (0.03, 1.38) | 0.20 (0.10, 0.42) |
| 7 | 0.39 (0.1,0 1.56) | 0.23 (0.11, 0.46) |
| 8 | 0.39 (0.10, 1.56) | 0.23 (0.11, 0.46) |
| 9 | 0.19 (0.03, 1.38) | 0.26 (0.13, 0.49) |
| 10 | 0 | 0.20 (0.10, 0.42) |
| 11 | 0.78 (0.29, 2.08) | 0.20 (0.10, 0.42) |
| 12 | 0.19 (0.03, 1.38) | 0.03 (0.00, 0.20) |

Abbreviations: CI, confidence interval.

**Supplemental eTable 6.** Adjusted Risk of Maternal Suicidal Behaviors During the First Postpartum Year in Mothers who Delivered via Cesarean Delivery, Compared with Mothers who Delivered Vaginally

|  | Adjusted Hazard Ratio (95% CI)^a^ | | |
| --- | --- | --- | --- |
|  | Any CD | Intrapartum CD^b^ | Planned CD^b^ |
| **Suicide attempt** | **1.57 (1.41, 1.75)** | **1.47 (1.22, 1.78)** | **1.74 (1.47, 2.06)** |
| **Death by Suicide** | 0.81 (0.43, 1.53) | 0.91 (0.27, 2.99) | 0.66 (0.16, 2.78) |

Abbreviation: CD, cesarean delivery; CI, confidence interval; VD, vaginal delivery.

^a^Model adjusted for calendar year at delivery, maternal age at delivery, highest education level, marital status/cohabitation, parity, history of suicide attempts, history of any psychiatric disorder and an interaction term for CD and country of birth.

^b^Analyses were performed in a sub-cohort of women who delivered between 1990-2012.

**Supplemental eTable 7.** Risk of Maternal Suicidal Behaviors During the First Postpartum Year in Mothers Who Delivered via Cesarean Delivery, compared with Mothers Who Delivered via Spontaneous Vaginal Delivery between 1990 and 2012

|  | Hazard Ratio (95% CI)^a^ | | | |
| --- | --- | --- | --- | --- |
|  | Any CD^b^ | Intrapartum CD | Planned CD | Instrumental VD |
| **Suicide attempt** | **1.43 (1.27, 1.61)** | **1.31 (1.12, 1.55)** | **1.54 (1.33, 1.80)** | 0.93 (0.76, 1.12) |
| **Death by suicide** | 1.23 (0.57, 2.67) | 0.95 (0.29, 3.09) | 1.46 (0.57, 3.72) | 1.54 (0.60, 3.95) |

Abbreviation: CD, cesarean delivery; CI, confidence interval; VD, vaginal delivery.

^a^ Model adjusted for calendar year at delivery.

^b^ Any CD includes intrapartum and planned CD.
